# Early Elevation of Complement Factor Ba Is a Predictive Biomarker for Transplant-Associated Thrombotic Microangiopathy

**DOI:** 10.3389/fimmu.2021.695037

**Published:** 2021-07-13

**Authors:** Hiroshi Okamura, Hirohisa Nakamae, Takero Shindo, Katsuki Ohtani, Yoshihiko Hidaka, Yasufumi Ohtsuka, Yosuke Makuuchi, Masatomo Kuno, Teruhito Takakuwa, Naonori Harada, Mitsutaka Nishimoto, Yasuhiro Nakashima, Hideo Koh, Asao Hirose, Mika Nakamae, Nobutaka Wakamiya, Masayuki Hino, Norimitsu Inoue

**Affiliations:** ^1^ Hematology, Graduate School of Medicine, Osaka City University, Osaka, Japan; ^2^ Hematology/Oncology, Kyoto University Graduate School of Medicine, Kyoto, Japan; ^3^ Department of Clinical Neutrition, Rakuno Gakuen University, Ebetsu, Japan; ^4^ The Japanese Association for Complement Research, Wakayama, Japan; ^5^ Department of Molecular Genetics, Wakayama Medical University, Wakayama, Japan; ^6^ Department of Pediatrics, Faculty of Medicine, Saga University, Saga, Japan; ^7^ Department of Medicine and Physiology, Rakuno Gakuen University, Ebetsu, Japan

**Keywords:** allogeneic hematopoietic stem cell transplantation, transplantation associated-thrombotic microangiopathy, complement, alternative pathway, Ba

## Abstract

Transplant-associated thrombotic microangiopathy (TA-TMA) is a fatal complication after allogeneic hematopoietic stem cell transplantation (allo-HSCT). Previous reports suggest that TA-TMA is caused by complement activation by complement-related genetic variants; however, this needs to be verified, especially in adults. Here, we performed a nested case-control study of allo-HSCT-treated adults at a single center. Fifteen TA-TMA patients and 15 non-TA-TMA patients, matched according to the propensity score, were enrolled. Based on a previous report showing an association between complement-related genes and development of TA-TMA, we first sequenced these 17 genes. Both cohorts harbored several genetic variants with rare allele frequencies; however, there was no difference in the percentage of patients in the TA-TMA and non-TA-TMA groups with the rare variants, or in the average number of rare variants per patient. Second, we measured plasma concentrations of complement proteins. Notably, levels of Ba protein on Day 7 following allo-HSCT were abnormally and significantly higher in TA-TMA than in non-TA-TMA cases, suggesting that complement activation *via* the alternative pathway contributes to TA-TMA. All other parameters, including soluble C5b-9, on Day 7 were similar between the groups. The levels of C3, C4, CH50, and complement factors H and I in the TA-TMA group after Day 28 were significantly lower than those in the non-TA-TMA group. Complement-related genetic variants did not predict TA-TMA development. By contrast, abnormally high levels of Ba on Day 7 did predict development of TA-TMA and non-relapse mortality. Thus, Ba levels on Day 7 after allo-HSCT are a sensitive and prognostic biomarker of TA-TMA.

## Introduction

Allogeneic hematopoietic stem cell transplantation (allo-HSCT) enables long-term survival of those with high-risk hematological disorders; however, there are some fatal treatment-related complications. Transplant-associated thrombotic microangiopathy (TA-TMA) is one of these, and the prognosis is miserable (1). In addition, diagnosis and management remain challenging (2). TA-TMA is characterized by systemic vascular endothelial injury, resulting in hemolytic anemia, consumptive thrombocytopenia, elevated lactate dehydrogenase levels, and schistocytes in peripheral blood (PB) smears. Ultimately, it causes renal failure and central nervous system disturbance, both of which are major causes of non-relapse mortality (NRM) following allo-HSCT (3).

Previous reports show that human leukocyte antigen (HLA)-mismatched donors, conditioning intensity, acute graft-versus-host disease (aGVHD), calcineurin inhibitors, and viral infection are risk factors for TA-TMA; thus TA-TMA is a multifactorial and heterogeneous disorder (4–6). Interestingly, recent studies indicate that the complement system may contribute to development of TA-TMA (7–12). First, a pediatric cohort suggested an association between genetic abnormalities of complement-related genes and TA-TMA (9). Rare variants (allele frequencies < 1%) of the 17 complement-related genes were more common in TA-TMA patients, suggesting that some genetic backgrounds predispose to TA-TMA. However, no similar studies of adult cases have been reported. Second, elevated levels of soluble terminal complement complex (sC5b-9) might be a predictive, diagnostic, and prognostic biomarker for TA-TMA (7, 8, 10, 12). Third, reports show that TA-TMA can be treated successfully with eculizumab, an anti-C5 antibody (13–16). Thus, increased activation of complement may cause TA-TMA; however, the detailed mechanisms underlying complement activation in TA-TMA are still unclear.

Here, we analyzed single nucleotide variants (SNVs) of complement-related genes, along with temporal changes in complement protein levels in TA-TMA and non-TA-TMA cases. We then examined the association between adult TA-TMA and the complement system, along with the significance of complement biomarkers for adult TA-TMA.

## Materials and Methods

### Study Design and Patient Selection

This was a retrospective nested case-control study. Consecutive patients who underwent allo-HSCT at Osaka City University Hospital between December, 2012 and December, 2016 were enrolled. Patients who did not consent, who developed uncertain TA-TMA, or for whom DNA samples were not available, were excluded. Uncertain TA-TMA was defined as the cases which could not exclude TA-TMA due to the absence of haptoglobin test and/or Coombs test or due to other causes of thrombocytopenia or hemolysis (e.g., drug, uncontrolled underlying hematological disease, or hemophagocytic syndrome). Next, patients with TA-TMA were distinguished from those without, and the propensity score (PS) for each patient was calculated. Based on this score, patients with TA-TMA were matched with those without TA-TMA (ratio, 1:1). Finally, the TA-TMA cohort and non-TA-TMA cohort were analyzed.

Variants of 17 genes related to complement pathways were examined in both groups, along with temporal measurement of complement proteins. This study was approved by the Ethical Committee of Osaka City University Graduate School of Medicine. The transplantation procedure is described in the **Supplementary Material**.

### Diagnostic Criteria for TA-TMA

TA-TMA was defined according to a previous report (17): [1] lactate dehydrogenase levels above the upper limit of normal; [2] *de novo* thrombocytopenia with a ≥ 50% decrease in the platelet count, or thrombocytopenia requiring transfusion support; [3] *de novo* progressive anemia or anemia requiring transfusion support; [4] histologic evidence of microangiopathy or a ≥ 1% presence of schistocytes in the PB; [5] absence of a coagulopathy; [6] haptoglobin level below the lower limit of normal; and [7] a negative Coombs test. The date of TA-TMA onset was defined as the first day on which all of the above criteria were fulfilled. We did not use two cells per high power field but 1% presence in 1000 red blood cells as the abnormal threshold value of a schistocyte count because we had only these qualitative results due to retrospective nature. International Council for Standardization in Haematology recommends this threshold for diagnosing thrombotic microangiopathic anemia (18).

### Genetic Analysis

Genomic DNA was purified from PB samples using a QIAamp DNA Blood Midi Kit (Qiagen, Valencia, CA). Target DNA fragments for sequencing were prepared using the SureSelect QXT Target Enrichment System (Agilent Technologies, Santa Clara, CA). Briefly, 50 ng of genomic DNA was digested enzymatically, ligated to adaptors, and amplified. The amplified DNA fragments were enriched for the exons of 136 complement-related genes using a biotinylated RNA capture library and streptavidin-conjugated magnetic beads (Agilent Technologies). Paired-end reads (150 bp) of the prepared DNA fragments were sequenced using a MiSeq Reagent Kit version 2 (300 cycles) and the MiSeq system (Illumina, San Diego, CA). The sequence reads were mapped to a human genome reference sequence (GRCh37/hg19) using the SureCall software program (version 2.1 or 3.5; Agilent Technologies), and single nucleotide variants were detected. In this study, variants of 17 genes (*C3, C5, CFB, CFD, CFH, CFI, CFP, C4BPA, CD46, CD55, CD59, THBD, CFHR1, CFHR3, CFHR4, CFHR5*, and *ADAMTS13*) of the 136 complement-related genes initially sequenced were analyzed (**Supplemental Table 2**) (9). Rare variants were selected from those variants with a minor allele frequency <1% in the Human genetic variation database of the Japanese population or the Genome Aggregation Database, which is composed of exome and genome sequence data from individuals worldwide. The pathogenicity of identified variants was analyzed using the Human gene mutation database and ClinVar, which aggregate information on the relationships between genomic variations and human phenotypes, and using tools that predict the pathogenicity of variants (i.e., Polyphen-2, SIFT, and PROVEAN).

### Complement Examination

Promptly after blood sampling, ethylene-diamine-tetra-acetic acid, disodium salt-treated plasma samples were obtained by centrifugation at 1,880 ×*g* for 20 minutes and stocked at -80°C at the Department of Clinical Examination, Osaka City University, until required. Nine complement tests (C3, C4, CH50, Ba, C5a, sC5b-9, CFI, CFH, and anti-CFH-antibody) were performed at four time points: at pre-conditioning, and at Days 7, 28, and 60 after allo-HSCT. Plasma CH50 levels were measured by hemolysis of sensitized sheep erythrocytes. The concentrations of C3 and C4 were measured by nephelometry (Nittobo Medical Co., Ltd., Tokyo, Japan). The concentrations of CFH, CFI, and anti-CFH autoantibodies were measured using ELISA kits (Abnova, Taipei, Taiwan). The concentrations of Ba, sC5b-9, and C5a were measured using MicroVue Ba EIA, MicroVue SC5b-9 Plus EIA, and MicroVue C5a EIA (Quidel, San Diego, CA) kits, respectively. We used the average levels ± 2 standard deviations of complement proteins in 70 Japanese healthy volunteers (age: 26-75 years) as the normal range (19).

### Propensity Score Matching

The PS for each patient was calculated using a multiple logistic regression model of TA-TMA development. Nine pre-transplant factors (reported as risk factors for TA-TMA) were used as variables: recipient age, sex, disease status (complete remission [CR] or non-CR), relationship to donor (related or non-related), HLA compatibility (matched or mismatched), graft source (bone marrow, peripheral blood [PB], or cord blood), conditioning intensity (myeloablative conditioning or reduced intensity conditioning), GVHD prevention (cyclosporine-based or tacrolimus-based), and the cytomegalovirus (CMV) serostatus of the recipient (seropositive or seronegative) (1, 8, 17, 19–22). Based on the PS, controls (non-TA-TMA) were matched (ratio, 1:1) with TA-TMA patients using a caliper within a standard deviation of 0.25. Patients who were alive with neither relapse nor TA-TMA when paired patients developed TA-TMA were selected as controls. We allowed matching with replacement if a control candidate matched by PS was excluded due to death or relapse of hematological disease when a paired patient developed TA-TMA.

### Statistical Analysis

The two cohorts were compared using the Fisher exact test for categorical variables and the Mann-Whitney U test for continuous variables. Student t test was used to compare complement protein levels between the two cohorts. The hazard ratio for TA-TMA development was calculated by cause-specific analysis using a Cox proportional hazards model. A restricted cubic spline, which can assess a non-linear relationship, was used to calculate the hazard ratio. The performance of complement protein levels at the early phase following allo-HSCT for predicting TA-TMA development was assessed using receiver-operating characteristic (ROC) curve analysis. An overall survival (OS) event was defined as death from any cause. NRM was defined as death without relapse/progression (Rel/Prog). Acute and chronic GVHD were diagnosed and graded according to established criteria (20, 21). Steroid refractory aGVHD (SR-aGVHD) was defined as grade 2–4 aGVHD that did not respond to standard prednisone therapy (1–2 mg/kg body weight) and which required second-line therapy. The diagnosis of CMV disease was established based on the presence of symptoms, clinical signs of organ damage reflected in laboratory tests and confirmed by biopsy (22). The probability of OS was calculated using the Kaplan–Meier method, and results were compared using the log-rank test. The cumulative incidences of GVHD, CMV disease, Rel/Prog and NRM were calculated using Gray’s method. Non-GVHD death was treated as a competing event for GVHD. Rel/Prog and NRM were treated as competing events.

All *P*-values and 95% confidence intervals (CIs) were two-sided. A *P*-value < 0.05 was deemed statistically significant. Analyses were performed using R version 3.5.1.

## Results

### Patients and Transplantation

**Figure 1:**
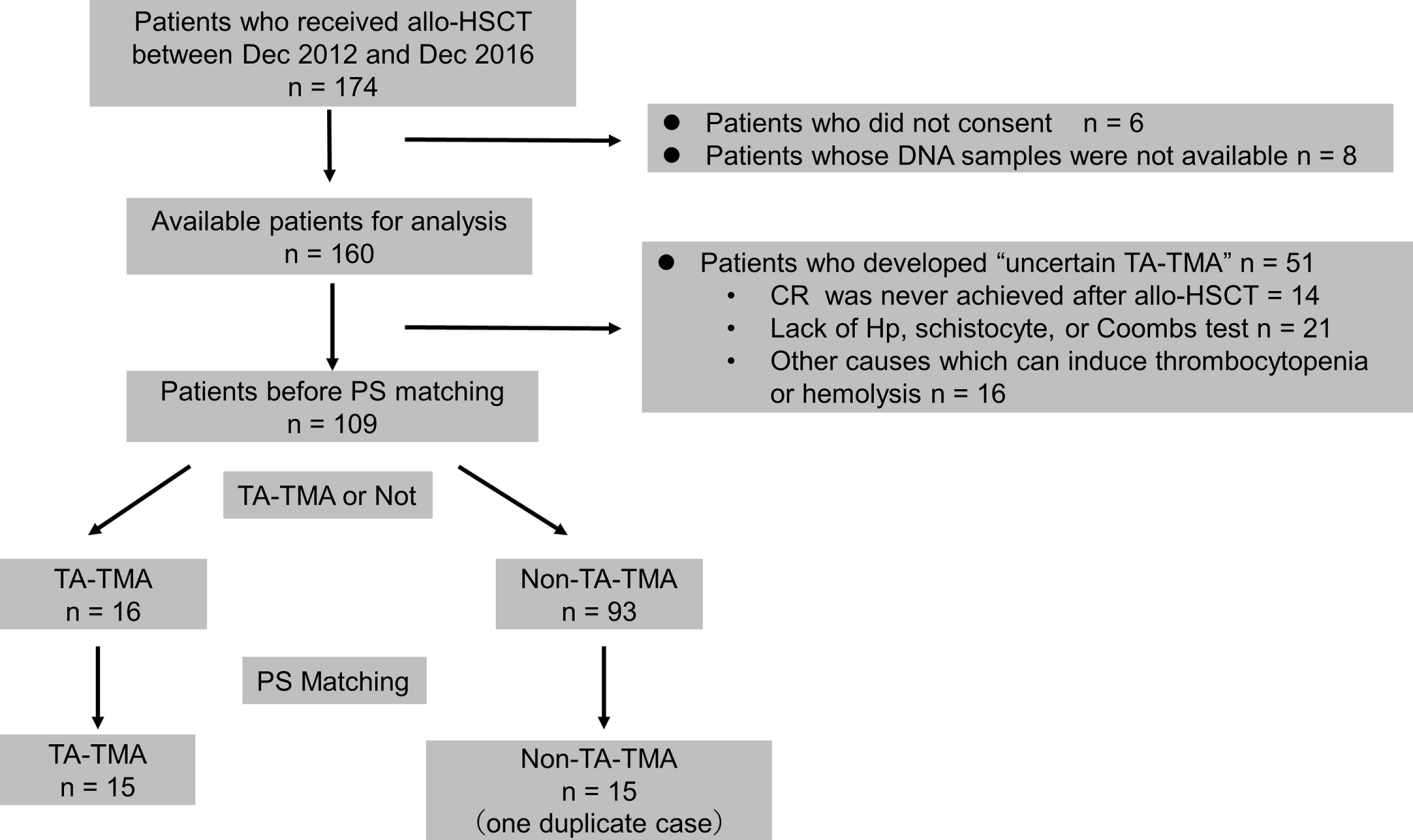
Flowchart for patient selection. allo-HSCT, allogeneic hematopoietic stem cell transplantation; CR, complete response; Hp, haptoglobin; PS, propensity score; TA-TMA, transplant-associated thrombotic microangiopathy.

A flowchart depicting patient selection is shown in **Figure 1**. One hundred-and-seventy-four patients underwent allo-HSCT. Six patients who did not consent, and eight patients for whom no DNA samples were available, were excluded, as were 51 patients who developed uncertain TA-TMA. Thus, 109 patients (TA-TMA, 16 patients; non-TA-TMA, 93 patients) were selected as candidates for PS matching. PS matching yielded 15 controls (non-TA-TMA) and 15 cases (TA-TMA), which were then analyzed. One of the controls was duplicated by matching with replacement and thus was analyzed twice (patient number 05025, **Supplemental Table 4**).

The median onset of TA-TMA was Day 25 following allo-HSCT (interquartile range: 22.5–44). There were no cases that administered eculizumab. Complement protein levels in one case of TA-TMA could not be measured before allo-HSCT due to the small volume of the plasma sample. Three TA-TMA lacked plasma samples at Day 60 following allo-HSCT due to early death (on Days 37, 44, and 57). The characteristics of the 106 patients before PS matching and of the 30 patients after PS matching are shown in **Table 1**. The detailed characteristics in TA-TMA patients are shown in **Supplemental Table 1**.

**Table 1:** Patient characteristics.

		Before PS matching			After PS matching		
		TA-TMA	non-TA-TMA	*P*-value	TA-TMA	non-TA-TMA*	*P*-value
n		16	93		15	15	
Median age [range]		54.5 [36–68]	46 [19–68]	0.005	55 [36–68]	58 [40–67]	0.59
Sex	male/female	8/8	54/39	0.59	8/7	8/7	1
Disease (%)				0.06			0.73
	AML	3 (19)	39 (42)		3 (20)	5 (33)	
	ALL	2 (13)	23 (25)		2 (13)	2 (13)	
	MDS	4 (25)	11 (12)		3 (20)	2 (13)	
	other	7 (44)	20 (22)		7 (46)	6 (40)	
Disease status	CR/non-CR	5/11	53/40	0.06	5/10	4/11	1
HLA disparity	match/mismatch/unknown	6/10/0	41/51/1	0.82	5/10/0	4/11/0	1
Conditioning intensity	MAC/RIC	3/13	48/45	0.016	3/12	4/11	1
Graft source (%)				0.94			0.41
	BM	5 (31)	28 (30)		4 (27)	4 (27)	
	PB	9 (56)	48 (52)		9 (60)	6 (40)	
	CB	2 (13)	17 (18)		2 (13)	5 (33)	
GVHD prophylaxis	CsA/Tac based	2/14	34/59	0.083	2/13	1/14	1
CMV sero-status in recipient	positive/negative	15/1	76/17	0.46	14/1	12/3	0.6

*including one duplicated patient.

AML, acute myeloid leukemia; ALL, acute lymphoblastic leukemia; BM, bone marrow; CB, cord blood; CMV, cytomegalovirus; CR, complete response; CsA, cyclosporine; GVHD, graft-versus-host disease; HLA, human leukocyte antigen; MAC, myeloablative conditioning; MDS, myelodysplastic syndrome; PB, peripheral blood; PS, propensity score; RIC, reduced intensity conditioning; Tac, tacrolimus; TA-TMA, transplant-associated thrombotic microangiopathy.

### No Association Between Complement-Related Genetic Variants and TA-TMA

In accordance with a previous report, we examined the association between TA-TMA and rare variants of 17 complement-related genes (**Supplemental Table 2**) (9). For the 30 patients with TA-TMA or non-TA-TMA, we found 35 rare variants in the Genome Aggregation Database (gnomAD): 17 non-synonymous variants, 11 synonymous variants, five intronic variants around the exon-intron boundaries, and two frameshift variants. No variants of four genes (*CD46, CD55, CD59*, and *CFP*) were identified. Sixteen variants showed allele frequencies < 1% in the Human genetic variation database of the Japanese (HGVD). These variants contained seven non-synonymous variants (including one variant with an allele frequency > 1% in gnomAD), six synonymous variants, two intronic variants, and one frameshift variants. All of these rare variants were heterozygous. The detailed results are shown in **Supplementary Tables 3**, **4**.

Three rare variants in TA-TMA patients in gnomAD have been reported previously. A variant in the *CFI* gene (p.R201S) was predicted to be a disease-associated polymorphism (DP), but was in fact a variant with a higher allele frequency (0.02233) in the HGVD. A variant of the *THBD* gene (p.D486Y) was reported as a causative mutation of atypical hemolytic uremic syndrome (aHUS), leading to dysregulation of complement activation (23, 24). However, this variant was classified as “DM?”, which has been registered as a likely disease-causing mutation but with questionable pathogenicity in the Human gene mutation database, and as a benign mutation in ClinVar. Although a variant in the *CFH* gene (p.V837I) was reported as a predisposing mutation in patients with aHUS (25), the effect of this mutation on gene function is unclear and this variant had a higher allele frequency (0.0183) in the HGVD. A variant in the *CFI* gene (p.R201S) was predicted to be a DP, but had a higher allele frequency (0.02233) in the HGVD. Two non-synonymous variants with an allele frequency < 1% in both the HGVD and gnomAD databases (p.V324A in *C4BPA* and p.R85H in *THBD*) were found in TA-TMA patients. *In silico* analysis predicted that the variant p.V324A in *C4BPA* was possibly damaging in Polyphen-2 (score, 0.680), damaging in SIFT (score, 0.043), and deleterious in PROVEAN (score, -3.14). The variant p.R85H in *THBD* was predicted to be possibly damaging in Polyphen-2 (score, 0.794) but tolerated in SIFT (score, 0.207) and neutral in PROVEAN (score, -2.02).

Analysis of the HGVD showed that 14 patients (46.7%) had at least one rare genetic variant (**Table 2**). Importantly, the percentage of patients in the TA-TMA (nine patients, 60.0%) and non-TA-TMA (five patients, 33.3%) groups with rare variants was not significantly different (*P* = 0.27). Non-synonymous rare variants were identified in all five non-TA-TMA patients but in only three of nine patients with TA-TMA. Rare synonymous variants and intronic variants were found only in patients with TA-TMA. HGVD revealed that the average number of rare variants per patient was 0.80 in the TA-TMA group and 0.33 in the non-TA-TMA group, but the average number of non-synonymous or frameshift variants was higher in non-TA-TMA patients (0.33) than in TA-TMA patients (0.27) (**Table 3**). These results suggest that the genetic background with respect to complement genes does not differ between TA-TMA and non-TA-TMA patients.

**Table 2:** Proportion of patients with rare variants.

Patient	Effect of variant	HGVD	gnomAD
TA-TMA (n = 15), n (%)	Non-synonymous or frameshift	3 (20.0)	5 (33.3)
	Synonymous	4 (26.7)	6 (40.0)
	Intronic variants	2 (13.3)	3 (20.0)
	Total	9 (60.0)	11 (73.3)
non-TA-TMA (n = 15), n (%)	Non-synonymous or frameshift	5 (33.3)	10 (66.7)
(including a duplicated patient)	Synonymous	0 (0.0)	7 (46.7)
	Intronic variants	0 (0.0)	2 (13.3)
	Total	5 (33.3)	12 (80.0)

gnomAD, Genome Aggregation Database; HGVD, Human genetic variation database of the Japanese population; TA-TMA, transplant-associated thrombotic microangiopathy.

**Table 3:** Average number of rare variants per patient.

Patient	Effect of variant	HGVD	gnomAD
TA-TMA (n = 15), n (range)	Non-synonymous or frameshift	0.27 (0–2)	0.73 (0–4)
	Synonymous	0.4 (0–2)	0.80 (0–4)
	Intronic variants	0.13 (0–1)	0.20 (0–1)
	Total	0.8 (0–2)	1.73 (0–8)
non-TA-TMA (n = 15), n (range)	Non-synonymous or frameshift	0.33 (0–1)	1.33 (0–4)
(including a duplicated patient)	Synonymous	0 (0)	0.60 (0–2)
	Intronic variants	0 (0)	0.13 (0–1)
	Total	0.33 (0–1)	2.1 (0–6)

gnomAD, Genome Aggregation Database; HGVD, Human genetic variation database of the Japanese population; TA-TMA, transplant-associated thrombotic microangiopathy.

### Abnormally High Levels of Ba on Day 7 Are Positively Associated With TA-TMA

**Figure 2:**
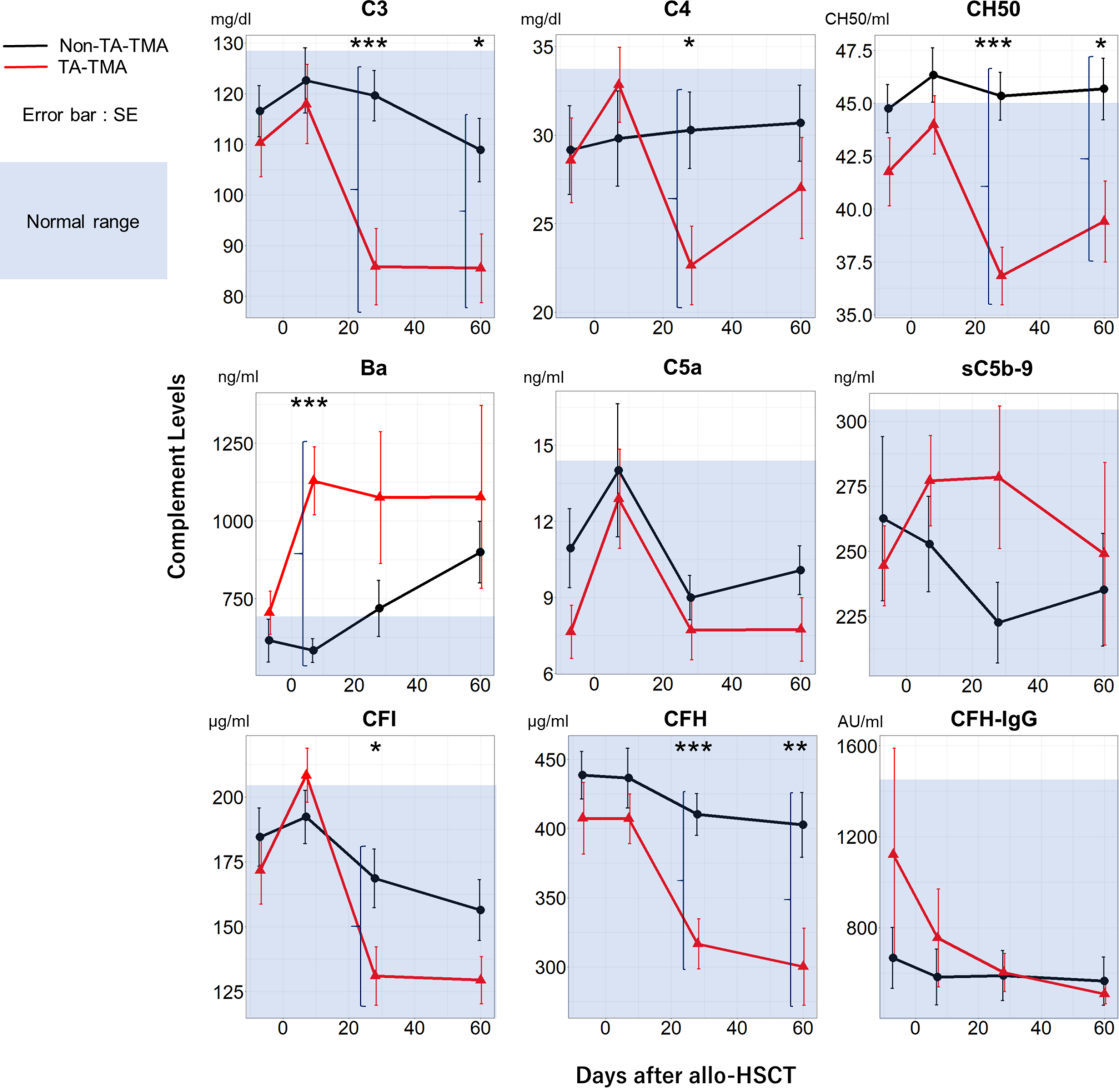
Temporal changes in complement protein levels in the TA-TMA and non-TA-TMA groups. Plasma levels of C3, C4, CH50, Ba, C5a, sC5b-9, CFI, CFH, and CFH-IgG at four time points (pre-conditioning, Day 7, Day 28, and Day 60) are shown for TA-TMA (red line) and non-TA-TMA (black line) patients. The blue shadow indicates the normal range in Japanese adults. *P < 0.05; **P < 0.01; ***P < 0.001. SE, standard error; TA-TMA, transplant-associated thrombotic microangiopathy.

Sequential measurement of complement proteins revealed several differences between the TA-TMA and non-TA-TMA groups (**Figure 2**). There were significant differences in Ba levels on Day 7 (*P* < 0.001), C3/C4/CH50/CFI/CFH on Day 28, and CH50/CFH on Day 60 following allo-HSCT. Notably, TA-TMA was characterized by abnormally high levels of Ba on Day 7 (mean ± standard error [SE]: 1129 ± 109 *vs.* 584 ± 38 ng/ml for non-TMA) and decreased C3/C4/CH50/CFI/CFH levels on Day 28. There were no differences in the levels of antibodies specific for CFH (one of the principal causes of aHUS) in either group.

**Figure 3:**
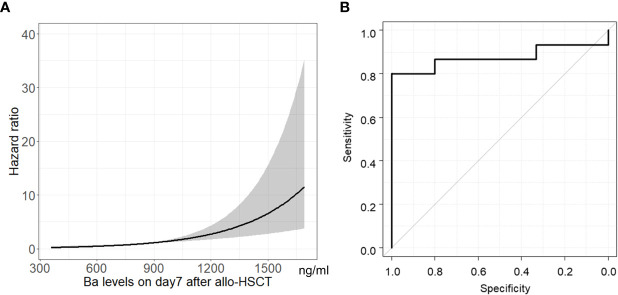
Association between Ba levels on Day 7 following allo-HSCT and TA-TMA development. Hazard ratio **(A)** and ROC curve **(B)** of Ba levels on Day 7 following allo-HSCT for TA-TMA development are shown. Gray shadow shows 95% CI of hazard ratio. allo-HSCT, allogeneic hematopoietic stem cell transplantation; CI, confidence interval; ROC, receiver-operating characteristic; TA-TMA, transplant-associated thrombotic microangiopathy.

The hazard ratio of Ba levels on Day 7 following allo-HSCT for TA-TMA development is shown in **Figure 3A**. As Ba levels increased, the hazard ratio for TA-TMA development increased. The area under the ROC curve (AUC) for Ba on Day 7 following allo-HSCT as a factor in TA-TMA development was 0.88 (95% CI, 0.72–1.00). The Youden index revealed that the best cut-off value for Ba was 869.1 ng/ml (**Figure 3B**), which had a positive predictive value (PPV) of 100% (95% CI, 64–100), a sensitivity of 80% (95% CI, 52–96), and a specificity of 100% (95% CI, 70–100).

### GVHD, Infection and the Prognosis According to Ba Levels During the Early Phase Following Allo-HSCT

Next, all analyzed patients were divided into high and low Ba groups using the above cut-off value for Ba (869.1 ng/ml) on Day 7 following allo-HSCT (which identifies TA-TMA development at the earliest time during the peri-transplant period). We compared the cumulative incidence of grade 2–4 aGVHD, SR-aGVHD, extensive chronic GVHD (cGVHD), and CMV disease between the two groups. Although the Ba levels on day 7 were not associated with cGVHD and CMV disease, it was associated with the incidence of aGVHD (*P* = 0.03, **Supplemental Figure 1**).

**Figure 4:**
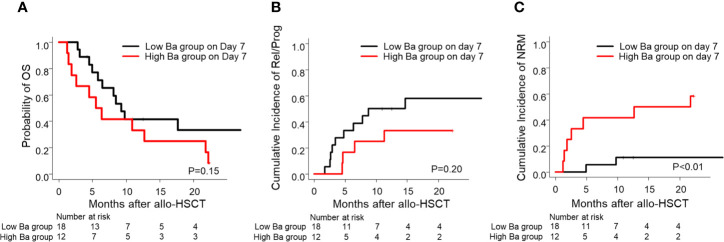
Prognosis of allo-HSCT in patients with low and high Ba levels on Day 7 following allo-HSCT. The probability of OS **(A)** and the cumulative incidence of Rel/Prog **(B)** and NRM **(C)** are shown for the groups with low Ba (< 869.1 ng/ml) and high Ba (≥ 869.1 ng/ml) on Day 7 following allo-HSCT. allo-HSCT, allogeneic hematopoietic stem cell transplantation; NRM, non-relapse mortality; OS, overall survival; Rel/Prog, relapse/progression.

Furthermore, we then compared the probability of OS and the cumulative incidence of Rel/Prog and NRM between the groups. Although we found no significant differences in the probability of OS and the cumulative incidence of Rel/Prog (**Figures 4A, B**, *P* = 0.15*, P* = 0.20), we found that the cumulative incidence of NRM in the high Ba group was higher than that in the low Ba group (**Figure 4C**, *P* < 0.01).

## Discussion

In this study, we examined rare variants of complement-related genes and temporal changes in the plasma concentrations of complement proteins in patients with TA-TMA and non-TA-TMA before and after allo-HSCT. We did not find an association between TA-TMA development and variants of 17 genes related to the complement system. However, we did find that Ba levels during the early phase following allo-HSCT are associated with not only TA-TMA development but also with NRM. These findings suggest that complement activation *via* the alternative pathway contributes to TA-TMA development, and that Ba levels during the early phase following allo-HSCT may be a novel predictive biomarker of TA-TMA development.

Jodele et al. reported that more complement gene variants were observed in patients with TA-TMA than in those without; however, our result is not consistent with their findings (9). This discrepancy could be due to differences in the characteristics of the patients analyzed. The participants in the study by Jodele et al. were pediatric patients, and the majority were not Asian. Furthermore, the major underlying diseases in their study were non-malignant disorders. All of the patients analyzed in our study were Japanese adults, and all had malignant hematological diseases. Although we found several rare non-synonymous variants (p.D486Y and p.R85H in *THBD* and p.V324A in *C4BPA*) of the 17 complement-related genes in TA-TMA patients, we did not find any variant that has been determined to cause aHUS. Further studies should clarify the functional and genetic association between these variants and TA-TMA development.

Previous studies report an association between TA-TMA and various complement proteins. Recently, Sartain et al. reported that Ba might be a diagnostic marker for TA-TMA in pediatric patients (11). They suggested that alternative pathway activation contributes to the pathogenesis of TA-TMA. However, Ba has never been identified as a useful biomarker that predicts TA-TMA development. Horváth et al. reported that elevated sC5b-9 levels on Day 28 following allo-HSCT can predict TA-TMA development (7). We also found that sC5b-9 levels in the TA-TMA group on Day 28 were higher than those in the non-TA-TMA group, but the differences were not statistically significant. Ba levels following allo-HSCT showed earlier and more significant changes in the TA-TMA and non-TA-TMA groups than sC5b-9 (**Figure 2**). Thus, Ba levels following allo-HSCT might be a more sensitive and earlier predictive biomarker for TA-TMA development than sC5b-9.

TA-TMA developed after Day 7 following allo-HSCT in all cases. Ba levels were elevated before TA-TMA development in all cases. This implies that the alternative pathway in TA-TMA cases was activated at an early time point following allo-HSCT. We did not have complement protein data on Day 60 for three of the TA-TMA cases due to early death. Therefore, we may have underestimated Ba levels on Day 60 in the TA-TMA group. Furthermore, C3, C4, CH50, CFI, and CFH levels in the TA-TMA group on Day 28 and/or 60 following allo-HCT were lower than those in the non-TA-TMA group, although they remained within the normal range (**Figure 2**). We speculate that complement activation in TA-TMA exhausts various complement proteins. The activation *via* the classical or lectin pathways, coupled with reduced levels of complement regulators, may lead to the activation of the alternative and terminal pathways. In our study, Ba was an earlier and more sensitive marker than C3 for the development of TA-TMA. The concentration of Ba in plasma is generally much less than that of C3. Therefore, Ba can be not only specific but also more sensitive for alternative pathway activation. It has been reported that multiple factors, including conditioning, infection, calcineurin inhibitor, and allo-reaction, can cause endothelial cell activation after allo-HSCT, which induces complement activation (26). We analyzed the association between Ba levels and the calcineurin inhibitor concentration on day 7 to detect the specific trigger of complement activation. However, no significant correlation was found (data not shown). Thus, we could not identify the trigger of complement activation, but it might derive from the combined effects of these multiple triggers of endothelial cell activation.

We found that the early abnormal elevation of Ba levels after allo-HSCT was associated with the cumulative incidence of grade 2–4 aGVHD (**Supplemental Figure 1**). Interestingly, all 12 patients in the high Ba group developed TA-TMA and 10 of whom developed grade 2–4 aGVHD. These findings suggest that early Ba elevation after allo-HSCT is associated with both TA-TMA and aGVHD. Recently, Wall and Li reported that the elevation of sC5b-9 and BBPlus in plasma at the onset or 2–6 weeks after the onset of aGVHD were high-risk factors for the subsequent development of TA-TMA (12, 27). Thus, the clinical course and phenotype in aGVHD with complement activation could differ from those in aGVHD without complement activation in terms of the subsequent development of TA-TMA. According to our findings, Ba might be predictive marker for aGVHD with TA-TMA. Clarifying causal relationships between aGVHD, TA-TMA, and complement activation is required for the development of an optimal treatment because the prognosis of TA-TMA with aGVHD is worse than that of aGVHD or TA-TMA alone (4, 28).

Furthermore, we found that early increases in Ba levels following allo-HSCT might be associated not only with TA-TMA development but also with an increased incidence of NRM. Recent studies show promising results for eculizumab as a treatment for TA-TMA (13–16). Furthermore, various anti-complement drugs, such as C3, C5, or *mannose-binding protein-associated serine protease 2 (MASP-2)* inhibitor have been developing recently. According to our findings, these anti-complement inhibitors are promising for TA-TMA treatment because any complement pathways (classical, lectin, alternative, and terminal pathways) may contribute to the development of TA-TMA. Thus, close monitoring, adjustment of immunosuppressive agents, or pre-emptive intervention using these complement inhibitors might be required for patients showing early elevation of Ba after allo-HSCT. The clinical trial of these complement inhibitors for patients with early abnormal Ba elevation after allo-HSCT is expected. In conclusion, our findings suggest that the alternative pathway is already activated before TA-TMA development, and that early elevation of Ba following allo-HCT is a novel predictive marker for TA-TMA related to NRM. Our findings are meaningful in that the present study represents the first exploratory research to show the significance of Ba as a predictive marker for the development of TA-TMA. However, as our research is retrospective one at a single affiliation, further study with a large and independent cohort is required to validate our findings.

## Data Availability Statement

The raw data supporting the conclusions of this article will be made available by the authors, without undue reservation.

## Ethics Statement

The studies involving human participants were reviewed and approved by Ethical Committee of Osaka City University Graduate School of Medicine. Written informed consent for participation was not required for this study in accordance with the national legislation and the institutional requirements.

## Author Contributions

HN, TS, YO, and NI designed the research. HO, YM, MK, TT, NH, MNi, YN, HK, HN, and MH acquired samples and data. MNa and AH constructed a database. YH, NI, and KO analyzed gene and complement protein. HO, HN, TS, NW, and NI analyzed and interpreted data. HO, HN, TS, and NI drafted and revised the paper. All authors critically reviewed the paper. All authors contributed to the article and approved the submitted version.

## Funding

This research was supported by a grant from the Japan Society for the Promotion of Science (JSPS) KAKENHI (number 20K07788 to MH and number JP17H04108 to NW, KO, and NI) and by a research grant from the Smoking Research Foundation (to NW). This research was also supported by the Japanese Association for Complement Research (JACR), which is funded by Alexion GK as company sponsored research. Alexion GK was not involved in the study design, collection, analysis, interpretation of data, the writing of this article or the decision to submit it for publication.

## Conflict of Interest

KO, NW, and NI are councilors of the Japanese Association for Complement Research (JACR). HN received research funds from Alexion GK.

The remaining authors declare that the research was conducted in the absence of any commercial or financial relationships that could be construed as a potential conflict of interest.
