# Supplementary material for: Early Elevation of Complement Factor Ba Is a Predictive Biomarker for Transplant-Associated Thrombotic Microangiopathy
Source: Front Immunol. 2021 Jul 13;12:695037. doi: 10.3389/fimmu.2021.695037 (PMC8315095; doi:10.3389/fimmu.2021.695037)
Supplement: Supplementary file 1 [file DataSheet_1.docx]

Supplementary Material

**Transplantation procedure**

In line with a previous report, a conditioning regimen containing either total body irradiation in fractionated doses greater than 8 Gy, an intravenous busulfan dose of 7.2 mg/kg or more, or a melphalan dose of 140 mg/m^2^ or more, was defined as myeloablative.(1) Human leukocyte antigen (HLA) compatibility was defined by DNA typing of HLA-A, HLA-B, HLA-C, and HLA-DR. Prophylactic antibiotics, either levofloxacin or polymyxin B tablets, an antifungal agent, and acyclovir were administered routinely from the start of conditioning. Trimethoprim-sulfamethoxazole was also administered from the start of conditioning up until 2 days prior to allogeneic hematopoietic stem cell transplantation (allo-HSCT) and after neutrophil engraftment to prevent *Pneumocystis jirovecii*-induced pneumonia. Granulocyte colony-stimulating factor treatment was initiated from the day after allo-HSCT, lasting up until neutrophil engraftment. Ursodeoxycholic acid was administered from the start of conditioning to prevent sinusoidal obstruction syndrome.

**Supplemental Table 1. Detailed characteristics in TA-TMA patients**

| **Patient**  **no.** | **Sex** | **Age**  **(years)** | **Diganosis** | **Disease Status** | **Conditioning regimen** | **Donor/Graft source** | **GVHD prophylaxis** | **Time from allo-HSCT to TA-TMA (day)** | **Plt count at　TA-TMA Diagnosis　(10,000/μl)** | **LDH level　at TA-TMA Diagnosis　(U/l)** | **Hb level at TA-TMA Diagnosis　(g/dl)** | **eGFR at TA-TMA Diagnosis　(ml/min/1.7)** | **Acute GVHD grade2 to 4 at TA-TMA Diagnosis** | **Active infection at TA-TMA Diagnosis** |
| --- | --- | --- | --- | --- | --- | --- | --- | --- | --- | --- | --- | --- | --- | --- |
| 05007 | F | 62 | ATLL | CR | Flu/Bu | MUD/BM | CsA/MTX | 64 | 2.3 | 803 | 6.9 | 123 | skin/grade2 | no |
| 05021 | M | 62 | MDS | non-CR | Flu/AraC/Mel | Haplo/PB | TAC/MMF/PTCY | 16 | 1.1 | 340 | 7.4 | 26 | gut,skin/grade2 | no |
| 05027 | F | 39 | AML | CR | Flu/AraC/Mel | Haplo/PB | TAC/MMF/PTCY | 365 | 2.4 | 522 | 7.9 | 45 | no | no |
| 05058 | M | 67 | MDS | non-CR | Flu/Bu | MUD/BM | TAC/MTX | 32 | 5.5 | 514 | 8.5 | 17 | no | BKV-HC |
| 05076 | F | 43 | ML | non-CR | MCNU/Flu/Mel | MRD/PB | CsA/MTX | 22 | 2.5 | 395 | 7.9 | 41 | no | no |
| 05081 | M | 60 | ML | non-CR | Flu/AraC/Mel | Haplo/PB | TAC/MMF/PTCY | 28 | 7.3 | 497 | 8.7 | 43 | gut,skin/grade2 | BKV-HC |
| 05087 | M | 55 | ATLL | non-CR | Flu/Mel/TBI | CB | TAC/MMF | 25 | 1.3 | 482 | 8.6 | 82 | skin,gut/grade2 | no |
| 05094 | M | 54 | MDS | non-CR | Bu/CY | MUD/BM | TAC/MTX | 56 | 0.6 | 273 | 7.1 | 71 | gut/grade3 | no |
| 05121 | F | 36 | ALL | CR | Flu/AraC/Mel | Haplo/PB | TAC/MMF/ATG | 10 | 3.2 | 897 | 7.8 | 59 | no | no |
| 05128 | M | 59 | AML | CR | Flu/AraC/Mel | Haplo/PB | TAC/MMF/PTCY | 23 | 2.8 | 267 | 9.5 | 107 | gut/grade3 | HHV6-encephalitis |
| 05144 | F | 49 | ML | CR | Flu/Mel | MUD/BM | TAC/MTX | 25 | 2.2 | 905 | 8.2 | 27 | gut/grade3 | no |
| 05147 | M | 68 | AML | non-CR | Flu/AraC/Mel | Haplo/PB | TAC/MMF/PTCY | 25 | 3.3 | 268 | 7.6 | 63 | gut/grade3 | no |
| 05158 | F | 47 | ML | non-CR | Flu/AraC/Mel | Haplo/PB | TAC/MMF/PTCY | 58 | 4.8 | 423 | 7.8 | 79 | no | no |
| 05161 | F | 51 | ALL | non-CR | Flu/AraC/Mel | Haplo/PB | TAC/MMF/PTCY | 27 | 0.1 | 424 | 8.5 | 58 | no | no |
| 05171 | M | 66 | ML | non-CR | Flu/Mel/TBI | CB | TAC/MMF | 18 | 1.7 | 281 | 8 | 62 | gut/grade3 | no |

Abbreviations: AML, acute myeloid leukemia; AraC, cytarabine; ALL, acute lymphoblastic leukemia; allo-HSCT, allogeneic hematopoietic stem cell transplantation; ATLL, adult T-cell leukemia/lymphoma; ATG, anti-thymocyte globulin; BKV, BK virus; BM, bone marrow; Bu, busulfan; CB, cord blood; CR, complete response; CsA, cyclosporine; F, female; Flu, fludarabine; CY, cyclophosphamide; eGFR, estimated glomerular filtration rate; GVHD, graft versus host disease; Haplo, haploidentical donor; Hb, hemoglobin; HC, hemorrhagic cystitis; HHV, human herpesvirus; LDH, lactate dehydrogenase; M, male; MCNU, methyl 6-[3-(2-chloroethyl)-3-nitrosoureido]-6-deoxy-alpha-D-glucopyranoside; MDS, myelodysplastic syndrome; Mel, melphalan; ML, malignant lymphoma; MMF, mycophenolate mofetil; MTX, methotrexate; MUD, matched unrelated donor; PB, peripheral blood; Plt, platelet; PTCY, post-transplant cyclophosphamide; TAC, tacrolimus; TA-TMA, transplant-associated thrombotic microangiopathy; TBI, total body irradiation;

**Supplemental Table 2. Genes tested**

| **Genes** | **Chromosome** | **Description** | **Accession no.** |
| --- | --- | --- | --- |
| *CFH* | 1 | Complement factor H | NM_000186.3 |
| *CFHR3* | 1 | Complement factor H-related 3 | NM_021023.5 |
| *CFHR1* | 1 | Complement factor H-related 1 | NM_002113.2 |
| *CFHR4* | 1 | Complement factor H-related 4 | NM_00121551.2 |
| *CFHR5* | 1 | Complement factor H-related 5 | NM_030787.3 |
| *C4BPA* | 1 | Complement component 4 binding protein, alpha | NM_00715.3 |
| *CD55* | 1 | CD55, decay accelerating factor | NM_000574.3 |
| *CD46* | 1 | CD46, membrane cofactor protein | NM_002389.4 |
| *CFI* | 4 | Complement factor I | NM_000204.4 |
| *CFB* | 6 | Complement factor B | NM_001710.6 |
| *C5* | 9 | Complement component 5 | NM_001735.2 |
| *ADAMTS13* | 9 | ADAM metallopeptidase with thrombospondin type 1 motif, 13 | NM_139026.4 |
| *CD59* | 11 | CD59 | NM_203330.2 |
| *CFD* | 19 | Complement factor D | NM_001928.3 |
| *C3* | 19 | Complement component 3 | NM_000064.2 |
| *THBD* | 20 | thrombomodulin | NM_000361.2 |
| *CFP* | X | Complement factor properdin | NM_002621.2 |

**Supplemental Table 3. Rare variants in TA-TMA patients**

| **Patient no.** | **Gene** | **Variant** | **Reference**  **SNP ID** | **MAF**  **in HGVD** | | **MAF**  **in gnomAD** | | **Annotation** | |
| --- | --- | --- | --- | --- | --- | --- | --- | --- | --- |
|  |  |  |  |  |  |  |  | **HGMD** | **ClinVar** |
| 05007 | *C5* | c.4763-9G>T | rs749681975 | ○ | ND | ○ | <0.00001 |  | NR |
| 05021 | *CFB* | p.G700 | rs116928087 |  | 0.02048 | ○ | 0.0004878 |  | B/LB |
|  | *CFH* | c.964+25C>T | rs56215062 | ○ | 0.00836 | ○ | 0.0004221 |  | NR |
| 05027 | *CFB* | p.F286 | rs117905900 |  | 0.02944 | ○ | 0.009972 |  | LB |
|  | *THBD* | p.E551 | N/A | ○ | ND | ○ | ND |  | NR |
| 05058 | *C4BPA* | p.V324A# | N/A | ○ | ND | ○ | ND |  | NR |
|  | *THBD* | p.D486Y | rs41348347 | ○ | 0.00748 | ○ | 0.007647 | DM?(2) | B |
| 05076 | *C4BPA* | p.P4Q | rs55867570 | ○ | 0.00342 |  | 0.03918 |  | NR |
| 05081 | *CFH* | p.V837I | rs55807605 |  | 0.0183 | ○ | 0.00138 | DM?(3) | B/LB |
|  | *CFHR3* | p.R142C | rs61737525 |  | 0.0166 | ○ | 0.007941 |  | B |
|  | *CFHR1* | p.Q242E | rs147253539 |  | 0.01907 | ○ | 0.008702 |  | NR |
|  | *CFI* | p.R201S | rs145769028 |  | 0.02233 | ○ | 0.0000795 | DP(4) | NR |
|  | *CFH* | p.T645 | rs56035657 |  | 0.01698 | ○ | 0.001396 |  | B/LB |
|  | *CFH* | p.G879 | rs55752475 |  | 0.01415 | ○ | 0.001392 |  | B/LB |
|  | *CFHR5* | p.A547 | rs74323799 | ○ | 0.00950 | ○ | 0.0001804 |  | VUS |
|  | *CFB* | p.S200 | rs113197809 | ○ | 0.00598 | ○ | 0.007758 |  | B/LB |
| 05087 | *C4BPA* | c.706+11A>G | rs79466538 |  | 0.02569 | ○ | 0.001279 |  | NR |
| 05094 | *C5* | p.D966Y | rs2230212 |  | 0.03897 | ○ | 0.004593 |  | NR |
|  | *C5* | p.R885H | rs56040400 |  | 0.01698 | ○ | 0.0003111 |  | A |
|  | *THBD* | p.R85H## | rs1462083784 | ○ | 0.00043 | ○ | <0.00001 |  | NR |
| 05121 | *CFHR5* | p.G450 | rs77159278 |  | 0.03595 | ○ | 0.0001233 |  | VUS |
| 05128 | *CFI* | p.R201S | rs145769028 |  | 0.02233 | ○ | 0.0000795 | DP(4) | NR |
|  | *CFHR1* | p.T80 | N/A | ○ | ND | ○ | ND |  | NR |
|  | *C3* | p.S541 | rs202078483 | ○ | 0.00718 | ○ | 0.001248 |  | B/LB |
| 05144 | *CFB* | p.F286 | rs117905900 |  | 0.02944 | ○ | 0.009972 |  | LB |
|  | *ADAMTS13* | p.T506 | rs782315134 | ○ | 0.00577 | ○ | 0.000043 |  | NR |
| 05158 | *C5* | p.D966Y | rs2230212 |  | 0.03897 | ○ | 0.004593 |  | NR |
| Abbreviations: MAF, minor allele frequency; ○, MAF<0.01; HGVD, Human genetic variation database; gnomAD, Genome Aggregation Database; N/A, not available; ND, no data; HGMD, Human gene mutation database; DM?, likely disease-causing mutation but with questionable pathogenicity; DP, disease-associated polymorphism; NR, not reported; B, benign; LB, likely benign; VUS, variants of uncertain significance; A, affected responsiveness to treatment with eculizumab  #p.V324A in *C4BPA* was predicted to be possibly damaging (Polyphen-2 score of 0.680), damaging (SIFT score of 0.043), or deleterious (PROVEAN score of -3.14).  ##p.R85H in *THBD* was predicted to be possibly damaging (Polyphen-2 score of 0.794), damaging (SIFT score of 0.043), or deleterious (PROVEAN score of -3.14). | | | | | | | | | |

**Supplemental Table 4. Rare variants in non-TA-TMA patients**

| **Patient no.** | **Gene** | **Variant** | **Reference**  **SNP ID** | **MAF**  **in HGVD** | | **MAF**  **in gnomAD** | | **Annotation** | |
| --- | --- | --- | --- | --- | --- | --- | --- | --- | --- |
|  |  |  |  |  |  |  |  | **HGMD** | **ClinVar** |
| 05015 | *ADAMTS13* | p.T339R | rs149517360 |  | 0.0338 | ○ | 0.002577 |  | B |
|  | *CFHR5* | p.G450 | rs77159278 |  | 0.03595 | ○ | 0.0001233 |  | VUS |
|  | *ADAMTS13* | c.1705+7G>A | rs78739717 |  | 0.03177 | ○ | 0.002755 |  | B |
| 05022 | *CFB* | p.G700 | rs116928087 |  | 0.02048 | ○ | 0.0004878 |  | B/LB |
| 05025* | *CFHR1* | p.D35fs*36 | N/A | ○ | 0.00306 | ○ | 0.00002972 |  | LOF |
| 05041 | *C5* | p.R885H | rs56040400 |  | 0.017 | ○ | 0.0003111 |  | A |
|  | *ADAMTS13* | p.G1181R | rs192619276 |  | 0.031 | ○ | 0.001780 |  | B |
| 05061 | *CFHR5* | p.G450 | rs77159278 |  | 0.03595 | ○ | 0.0001233 |  | VUS |
| 05107 | *CFH* | p.D798N# | rs55931547 | ○ | 0.00087 | ○ | 0.0000199 |  | NR |
|  | *C3* | c.600-14C>T | rs3745558 |  | 0.02808 | ○ | 0.005119 |  | B |
| 05111 | *CFH* | p.V837I | rs55807605 |  | 0.0183 | ○ | 0.001380 | DM?(3) | B/LB |
|  | *CFHR3* | p.R142C | rs61737525 |  | 0.0166 | ○ | 0.007941 | DM?(5) | B |
|  | *CFHR1* | p.Q242E | rs147253539 |  | 0.0195 | ○ | 0.008702 |  | NR |
|  | *ADAMTS13* | p.S903L | rs78977446 |  | 0.0439 | ○ | 0.003409 | DM | B/LB |
|  | *CFH* | p.T645 | rs56035657 |  | 0.01698 | ○ | 0.001396 |  | B/LB |
|  | *CFH* | p.G879 | rs55752475 |  | 0.01415 | ○ | 0.001392 |  | B/LB |
| 05115 | *CFH* | p.V837I | rs55807605 |  | 0.0183 | ○ | 0.001380 | DM?(3) | B/LB |
|  | *CFHR3* | p.R142C | rs61737525 |  | 0.0166 | ○ | 0.007941 | DM?(5) | B |
|  | *CFHR1* | p.Q242E | rs147253539 |  | 0.0195 | ○ | 0.008702 |  | NR |
|  | *ADAMTS13* | p.R558W## | rs782055634 | ○ | 0.00043 | ○ | 0.000052 |  | NR |
|  | *CFH* | p.T645 | rs56035657 |  | 0.01698 | ○ | 0.001396 |  | B/LB |
|  | *CFH* | p.G879 | rs55752475 |  | 0.01415 | ○ | 0.001392 |  | B/LB |
| 05136 | *CFH* | p.Y1058H | rs55679475 |  | 0.01224 | ○ | 0.0006296 | DM?(3) | LB |
|  | *CFH* | p.V1060L | rs55771831 |  | 0.01323 | ○ | 0.0005801 | DM?(3) | B/LB |
|  | *C5* | p.R885H | rs56040400 |  | 0.01698 | ○ | 0.0003111 |  | A |
|  | *CFB* | p.F286 | rs117905900 |  | 0.02944 | ○ | 0.009972 |  | LB |
| 05148 | *CFD* | p.D249N### | N/A | ○ | 0.00043 | ○ | ND |  | NR |
| 05156 | *CFHR4* | p.I527fs*7 | N/A |  | 0.01907 | ○ | <0.00001 |  | LOF |
|  | *ADAMTS13* | p.S903L | rs78977446 |  | 0.0439 | ○ | 0.003409 | DM | B/LB |
|  | *CFB* | p.F286 | rs117905900 |  | 0.02944 | ○ | 0.009972 |  | LB |
| *, duplicated patient matched by propensity score.  Abbreviations: MAF, minor allele frequency; ○, MAF<0.01; HGVD, Human genetic variation database; gnomAD, Genome Aggregation Database; N/A, not available; ND, no data; HGMD, Human gene mutation database; DM, disease-causing mutation; DM?, likely disease-causing mutation but with questionable pathogenicity; LOF, loss-of-function; NR, not reported; B, benign; LB, likely benign; VUS, variants of uncertain significance; A, affected responsiveness to treatment with eculizumab  #p.D798N in *CFH* was predicted to be benign (Polyphen-2 score of 0.218), tolerated (SIFT score of 0.150), or deleterious (PROVEAN score of -2.69).  ##p.R558W in *ADAMTS13* was predicted to be probably damaging (Polyphen-2 score of 0.999), damaging (SIFT score of 0.003), or deleterious (PROVEAN score of -5.67).  ###p.D249N in *CFD* was predicted to be benign (Polyphen-2 score of 0.002), tolerated (SIFT score of 0.062), or neutral (PROVEAN score of -0.59). | | | | | | | | | |

**Supplemental Figure 1. aGVHD, SR-aGVHD, extensive cGVHD, and CMV disease according to Ba levels during the early phase following allo-HSCT**


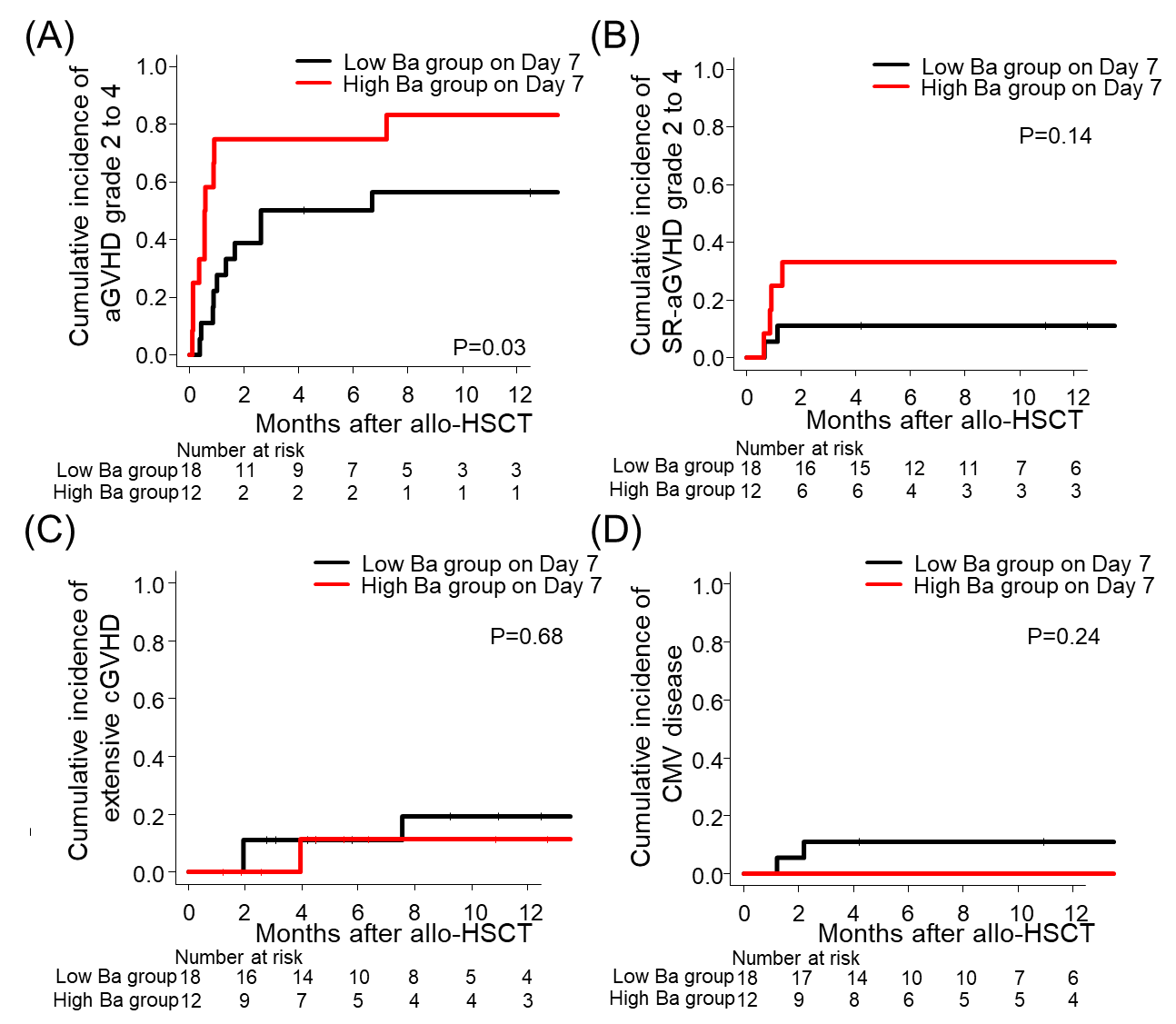


Abbreviations: aGVHD, acute graft versus host disease; allo-HSCT, allogeneic hematopoietic stem cell transplantation; cGVHD, chronic graft versus host disease; CMV, cytomegalovirus; SR-aGVHD, steroid refractory aGVHD.

The cumulative incidence of grade 2–4 aGVHD (A), SR-aGVHD (B), extensive cGVHD, and CMV disease in the low and high Ba groups on Day 7 after allo-HSCT.

**References to Supplementary Material**

1. Giralt S, Ballen K, Rizzo D, Bacigalupo A, Horowitz M, Pasquini M, et al. Reduced-intensity conditioning regimen workshop: defining the dose spectrum. Report of a workshop convened by the center for international blood and marrow transplant research. Biol Blood Marrow Transplant. 2009;15(3):367-9.

2. Delvaeye M, Noris M, De Vriese A, Esmon CT, Esmon NL, Ferrell G, et al. Thrombomodulin mutations in atypical hemolytic-uremic syndrome. N Engl J Med. 2009;361(4):345-57.

3. Matsumoto T, Fan X, Ishikawa E, Ito M, Amano K, Toyoda H, et al. Analysis of patients with atypical hemolytic uremic syndrome treated at the Mie University Hospital: concentration of C3 p.I1157T mutation. Int J Hematol. 2014;100(5):437-42.

4. Yuasa I, Nakagawa M, Umetsu K, Harihara S, Matsusue A, Nishimukai H, et al. Molecular basis of complement factor I (CFI) polymorphism: one of two polymorphic suballeles responsible for CFI A is Japanese-specific. J Hum Genet. 2008;53(11-12):1016-21.

5. Zhang T, Lu J, Liang S, Chen D, Zhang H, Zeng C, et al. Comprehensive Analysis of Complement Genes in Patients with Atypical Hemolytic Uremic Syndrome. Am J Nephrol. 2016;43(3):160-9.
